# Lignin Hydrogenolysis: Phenolic Monomers from Lignin and Associated Phenolates across Plant Clades

**DOI:** 10.1021/acssuschemeng.3c01320

**Published:** 2023-06-28

**Authors:** Mingjie Chen, Yanding Li, Fachuang Lu, Jeremy S. Luterbacher, John Ralph

**Affiliations:** † Department of Energy, Great Lakes Bioenergy Research Center, Wisconsin Energy Institute, Madison, Wisconsin 53726, United States; ‡ Institute of Chemical Sciences and Engineering, École Polytechnique Fédérale de Lausanne, Lausanne 1015, Switzerland; § Department of Biochemistry, University of Wisconsin-Madison, Madison, Wisconsin 53706, United States

**Keywords:** reductive catalytic fractionation (RCF), biomass, commodity chemical, lignin composition, DFRC, NMR, saponification

## Abstract

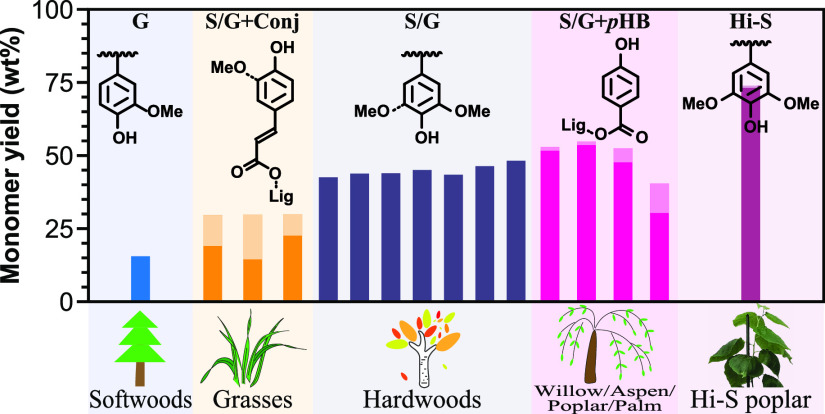

The chemical complexity of lignin remains a major challenge for lignin valorization into commodity and fine chemicals. A knowledge of the lignin features that favor its valorization and which plants produce such lignins can be used in plant selection or to engineer them to produce lignins that are more ideally suited for conversion. Sixteen biomass samples were compositionally surveyed by NMR and analytical degradative methods, and the yields of phenolic monomers following hydrogenolytic depolymerization were assessed to elucidate the key determinants controlling the depolymerization. Hardwoods, including those incorporating monolignol *p*-hydroxybenzoates into their syringyl/guaiacyl copolymeric lignins, produced high monomer yields by hydrogenolysis, whereas grasses incorporating monolignol *p*-coumarates and ferulates gave lower yields, on a lignin basis. Softwoods, with their more condensed guaiacyl lignins, gave the lowest yields. Lignins with a high syringyl unit content released elevated monomer levels, with a high-syringyl polar transgenic being particularly striking. Herein, we distinguish phenolic monomers resulting from the core lignin vs those from pendent phenolate esters associated with the biomass cell wall, acylating either polysaccharides or lignins. The basis for these observations is rationalized as a means to select or engineer biomass for optimal conversion to worthy phenolic monomers.

## Abstract 2

Hydrogenolytic depolymerization of lignin from 16 biomass samples shows the significant impact of lignin composition on phenolic monomer yields.

## Introduction

**Figure 1:**
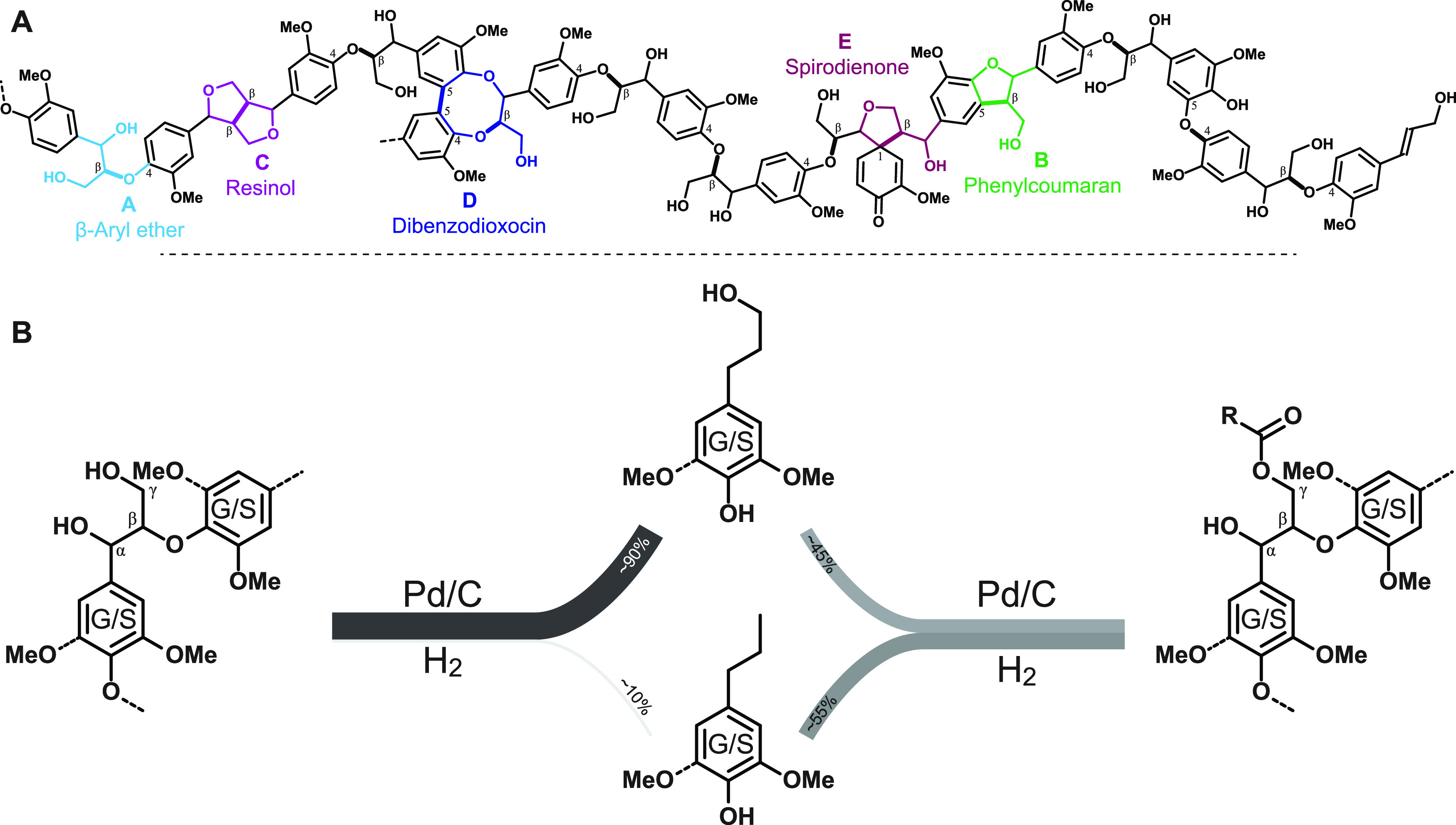
(A) A guaiacyl lignin model showing the main types of intermonomeric linkages, the β-aryl ether (β–O–4, **A**), and the C–C-linked units: phenylcoumaran (β–5, **B**), resinol (β–β, **C**), dibenzodioxocin (5–5/4–O−β, **D**), and spirodienone (β–1, **E**); a coniferyl alcohol end group resulting from initial dimerization is also shown starting from the right-hand end. (B) Hydrogenolysis (RCF, reductive catalytic fractionation) of β-ether units in lignin releases phenolic monomers. Selectivity for arylpropanol (top) over arylpropyl (bottom) products is ∼90:10 when using Pd/C-H_2_ and roughly reversed for Ru/C.^16,35,71,88,89^ If the γ-OH is acylated (right), as in grasses by *p*-coumarate and in certain hardwoods by *p*-hydroxybenzoate (and, not discussed, in certain other biomass plants by acetate), hydrogenolysis to the arylpropane is more prevalent^38^ [calculation: 100% γ-OH produces ∼90:10, 10% γ-ester produces ∼80:20, ⇒ 100% γ-ester produces ∼45:55; this should be determined more accurately using γ-acylated-β-ether model compound hydrogenolysis]. G: guaiacyl; S: syringyl. Minor *p*-hydroxyphenyl units (H, not shown) are largely ignored here.

**Figure 2:**
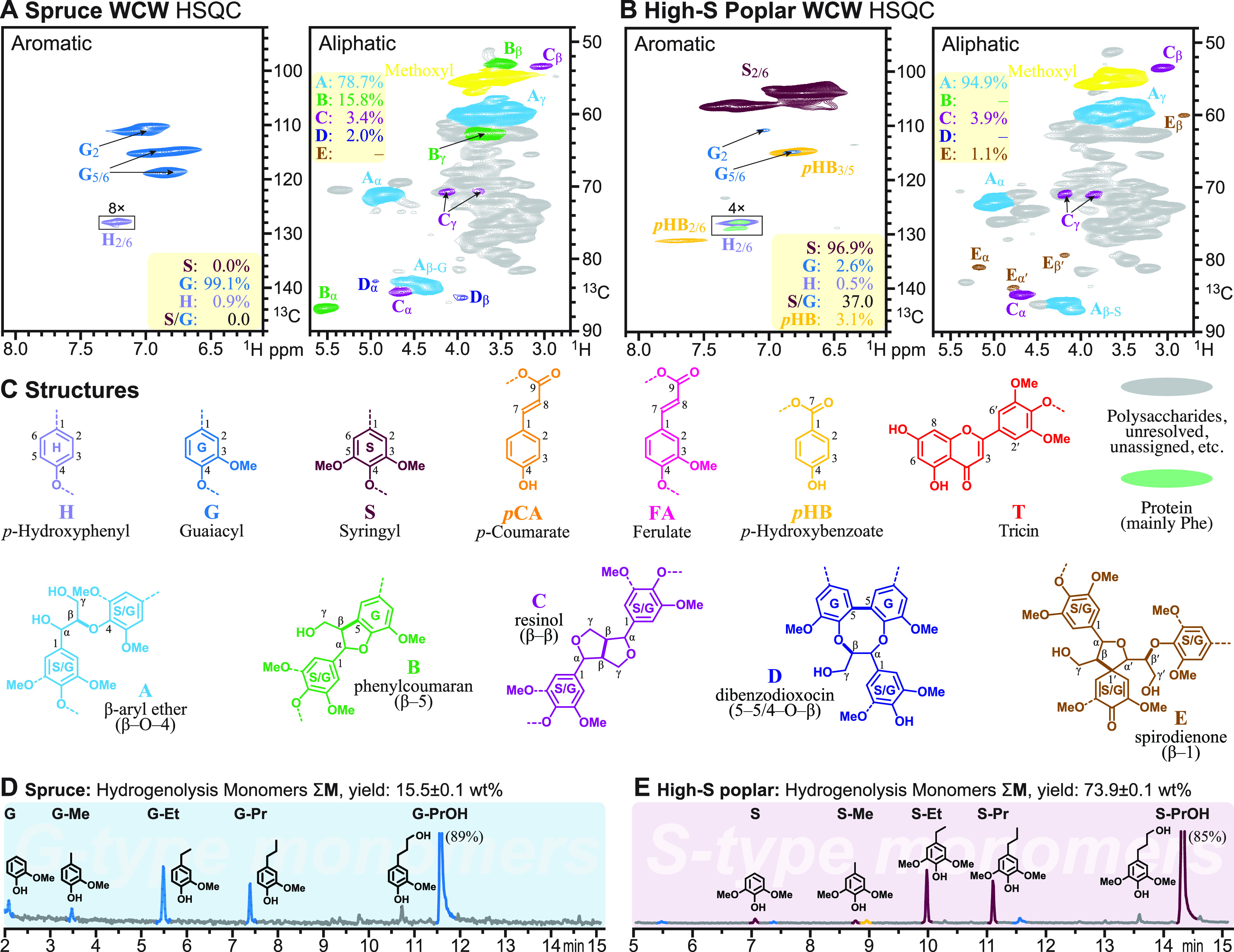
NMR analysis and hydrogenolytic monomer profiles from biomass that contains an essentially G-only lignin (spruce) and an essentially S-only lignin (high-S transgenic poplar), along with structures relevant for all figures. (A) Aromatic and oxygenated-aliphatic regions of an HSQC NMR spectrum of spruce whole-cell-wall material. (B) Analogous spectra from high-S poplar whole-cell-wall material. (C) Chemical structures and abbreviations used throughout (including structures not in the spruce or poplar here), color-coded as for (resolved) contours in the spectra. (D) GC-FID of hydrogenolytic monomers, produced in 15.5% yield based on Klason lignin, from spruce; using Pd/C as catalyst produces guaiacylpropanol as the major product (89 wt %). (E) GC-FID of hydrogenolytic monomers, produced in 73.9 wt % yield based on Klason lignin, from the high-S poplar; using Pd/C as catalyst produces syringylpropanol as the major product (85 wt %).

Lignin, a natural phenylpropanoid polymer, is one of the major components of lignocellulosic biomass, accounting for 15–35 wt % of dried biomass that is estimated to be available at the rate of 243–767 million tons per year by 2030 in the United States.^1^ Depolymerization of this sustainable, green, and abundant bioresource to provide valuable aromatic chemicals is therefore attracting increasing interest. Current depolymerization methods rely heavily on cleaving lignin’s most labile ether linkages (Figure 1).^2−4^ A major challenge originates from lignin’s intrinsic heterogeneity. Lignification is the process of polymerization from 4-hydroxyphenylpropanoids, primarily the monolignols (ML) *p*-coumaryl alcohol, coniferyl alcohol, and sinapyl alcohol, by combinatorial radical coupling reactions in the plant cell wall. Lignification produces guaiacyl **G** and syringyl **S** units in a polymer that has no specific sequence;^5,6^*p*-hydroxyphenyl **H** units (Figure 2) are typically minor (∼1%). The monomer-derived units in the polymer are characterized by the various types of interunit linkages between the monomeric units (Figure 1A): β-ethers **A** (or β-aryl ethers) from β–O–4-coupling, phenylcoumarans **B** from β–5-coupling, resinols **C** from β–β-coupling, biphenyls from 5–5-coupling that usually add a further monolignol by 4–O−β-coupling to produce dibenzodioxocins **D**, spirodienones **E** from β–1-coupling (of a monolignol with a preformed β-ether unit), and other more minor units.^6^ Adding to the complexity, some important bioenergy plants also utilize monolignol *p*-hydroxybenzoate (ML-*p*HB), monolignol *p*-coumarate (ML-*p*CA), monolignol ferulate (ML-FA), and other conjugates as “monomers” in lignification (Figure 2).^7−9^ Grasses even have polysaccharides that are acylated by *p*CA and FA.^10^ Such phenolic acids (PAs) can influence the monomer yields and obfuscate analyses of lignin depolymerization.^11^ We have been careful to separate these components in the analyses herein.

Few studies have reported monomer yields higher than 50 wt % of the lignin even with state-of-the-art methods such as reductive catalytic fractionation (RCF) and “lignin-first” methods that take advantage of the native lignin in biomass as opposed to the often condensed and degraded lignins deriving from industrial processes.^4,12,13^ Under the conditions used, simple hydrolysis reactions are responsible for cleaving β-ethers, liberating components including the monolignols themselves that are then stabilized by hydrogenation in the presence of the catalyst and H_2_.^14^ Hydrogenolysis refers to the cleavage reactions and hydrogen substitution that take place in addition to direct hydrogenation. We include the hydrolysis/stabilization reactions with the hydrogenolysis, perhaps both encapsulated in the term reductive catalytic fractionation (RCF). The process cleaves various ether linkages in lignin, including conventional β-ethers with their characteristic β–O–4 interunit linkages (Figure 1) but also the α,β-ethers in benzodioxane structures in special lignins derived from catecholic monomers and, to some degree, 5–O–4 linkages in minor biphenyl ether units.^14−17^ Assuming that the distribution of ether and C–C linkages within a linear lignin polymer is statistically weighted but otherwise random, the monomer yield from any ether-cleaving degradative reaction can be predicted by the following model:^5,18,19^
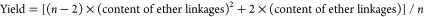
1in which *n* is the degree of polymerization of lignin. Ether linkages are those composed of only ether bonds and not C–C bonds, e.g., β–O–4. According to this model, it is logical that increasing the content of cleavable linkages in lignin will improve the monomer yield from lignin depolymerization processes. The dependence of hydrogenolytic monomer yield on ether linkage levels in lignin has been elucidated in prior research.^20,21^

Recent interest in enhancing lignin utilization, in part driven by the realization that lignin is the largest sustainable source of phenolics on the planet, has prompted new investigations into both lignin depolymerization and lignin biosynthesis in the plant. In the latter, misregulation of lignin monomer biosynthesis has been studied for its effect on the ultimate production of the polymer through lignification and has even progressed to attempts to design lignins for improved biomass processing, as we have touted and reviewed.^8,22^ An ambitious example of the latter approach was our attempt to produce new lignin polymers containing readily chemically cleavable linkages in the polymer backbone by inducing plants to utilize monolignol ferulate conjugates as lignin “monomers”.^23^ Once analytical methods had been developed to detect the introduction of monolignol ferulates into the lignin polymer, we discovered that nature was already utilizing such conjugates at low levels in its lignification in some plant lines.^23,24^

Even without delving into lignin engineering, it is well known that the various plant sources may produce markedly different lignins with respect to the component monomers and the resulting interunit linkage distribution.^25^ Factors other than the monomeric composition, as assessed by the syringyl/guaiacyl (S/G) ratio, appear to affect phenolic monomer yields, however.^26^ It is therefore anticipated that some plants can produce lignins that are more amenable to chemical cleavage to monomers than others. Gaining a knowledge of what kinds of plants produce such lignins will aid in our selection or engineering of plants to produce lignins that are more ideally suited for current depolymerization processes.

To understand the plant cell wall factors influencing phenolic monomer yield, 16 biomass samples, covering softwoods, hardwoods, and grasses, were collected and subjected to depolymerization studies by hydrogenolysis/RCF. These samples were classified into four groups.

(1) Gymnosperms/softwoods: As represented by a spruce sample, gymnosperm lignins are G-rich (and essentially G-only). The reason for using only a single sample in this class, as justified further below, is that G-lignins are quite invariant.
(2) Angiosperms/hardwoods/dicots: a group of conventional hardwoods, along with kenaf, with lignin composed of both G and S units.
(3) Hardwoods incorporating monolignol *p*-hydroxybenzoate (ML-*p*HB) conjugates: This group includes species from Salicaceae and Palmae families. Lignins in this group have G and S units as well as units derived from monolignol *p*-hydroxybenzoate (ML-*p*HB) conjugates,^27^ requiring that we carefully delineate monomer yields from the “core lignin” vs these easily released pendent units; we define the core lignin as that polymer chain resulting from the lignification of monolignol monomers or monolignol moieties (in conjugates), a term that was used in the past,^28^ has gone out of favor, but rather aptly describes the key polymer fraction required here. In this group, we also include a transgenic poplar engineered to be extremely S-rich;^29,30^ we compare it, though, with softwoods as another example of a single-monomer-derived lignin. Unfortunately, we do not have such high-S materials from any of the hardwoods in group 2 that are composed solely of S and G units and do not possess phenolate conjugates.
(4) Monocots/grasses: This group includes three typical monocot biomass samples, switchgrass, maize (corn stover), and wheat. Their lignins are again primarily G-S lignins but with units also derived from monolignol *p*-coumarate (ML-*p*CA) and monolignol ferulate (ML-FA) conjugates, again requiring that we carefully delineate monomer yields from the core lignin from these easily released pendent units.^7^ They additionally have arabinoxylan polysaccharides acylated with *p*CA and FA, implying that various phenolics produced by degradative methods do not solely derive from lignin. Incidentally, this has not gone unnoticed in the RCF field,^31^ but we stress it here as the origin of hydroxycinnamates often remains conflated in the literature.

These biomass samples cover the range of plants expected at a biorefinery operation. The intent is to document and rationalize the depolymerization performance of each class by hydrogenolysis and to aid in the selection, breeding, or engineering of plants for ready conversion into valuable phenolic monomers.

## Materials and Methods

### General

Chemicals and solvents were sourced from Sigma-Aldrich (St. Louis, MO, USA) unless otherwise noted.

### Biomass Materials

Biomass samples were preground into fine powder (<100 mesh) using a Retsch (Newtown, PA, USA) MM400 mixer mill with corrosion-resistant stainless steel screw-top grinding jars (50 mL) containing a single stainless steel ball bearing (30 mm). The preground biomass (∼1.5 g) was sequentially extracted using 40 mL of solvent under sonication for 30 min, repeating three times, with distilled water, 80% ethanol, and acetone.

Norway spruce (*Picea abies*), white birch (*Betula papyrifera*), and wheat straw (*Triticum aestivum*) were purchased from Amazon; red maple (*Acer rubrum*) was that used in previous studies;^32^ walnut (*Juglans major*), white oak (*Quercus alba*), and beech (*Fagus grandifolia*) were from John Harkin’s collection from the US Forest Products Laboratory; willow (*Salix babylonica*) was from 2-year-old branches from trees on the shores of Lake Mendota, Madison, WI; the poplar was an NM6 hybrid poplar (*Populus maximowiczii* × *nigra*); big-tooth aspen (*Populus grandidentata*) was from ∼30-year-old trees from Dane County, WI; the high-S poplar was a transgenic produced by Chapple et al., as previously described,^29,30,33,34^ and was the same material used in a prior study;^35^ palm empty fruit bunch material was that used in a prior study;^27^ corn stover (*Zea mays*) was the line previously used;^36−38^ the energy sorghum (*Sorghum bicolor*), switchgrass (*Panicum virgatum*), balsa (*Ochroma pyramidale*), and kenaf (*Hibiscus cannabinus*) were the same materials used previously,^24,36,39^ except that the kenaf core and not the bast fiber was used.

### Klason Lignin

Klason lignin of biomass was determined according to the NREL method.^40^ Biomass (∼150 mg, recorded as *M*_biomass_) was treated with 3 mL of 72 wt % sulfuric acid at 30 °C for 1 h. The solution was diluted by adding 84 mL of deionized water and heated at 121 °C for 1 h. The hydrolysate was cooled to room temperature and filtered with G3 sintered glass crucible. After drying at 105 °C, the crucible plus biomass residue was weighed (*M*_105_ in eq 2 below). The crucibles plus residue were then placed in a muffle furnace at 575 °C for 24 h, and the weight of the crucible plus ash was recorded (*M*_575_). Klason lignin was calculated as follows:
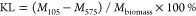
2

### Hydrogenolysis (RCF)

Hydrogenolysis of biomass was performed using methods reported in a previous study.^17^ Biomass (100 mg), catalyst (10 mg, 5 wt % Pd/C), and methanol (20 mL) were loaded into a 50 mL high-pressure Parr (Parr Instrument Company Moline, IL, USA) reactor. The reactor was purged five times with hydrogen and then pressurized to 40 bar at room temperature. Under mechanical stirring (700 rpm), the reactor was heated to 200 °C over 30 min, held for 3 h, and then cooled to room temperature. The resultant liquid was filtered through a Whatman polyamide membrane (0.45 μm pore size) and washed with methanol. The solvent (methanol) was removed under reduced pressure at 40 °C using a rotary evaporator. Monomer identity was validated by GC–MS. The crude products were dissolved in acetone and made up to 10 mL in a volumetric flask for GC-FID-based quantitative analysis using a 15 m, 0.25 mm ID, 0.25 μm Restek Rxi-5Sil MS fused silica GC column. Quantification of monomers was via calibration curves created using external standards, including guaiacyl monomers (Figure 2D) and syringyl monomers (Figure 2E), along with methyl *p*-hydroxybenzoate, methyl *p*-coumarate, and methyl ferulate. The GC program was as follows: injector 250 °C, split ratio 20:1, heat column at 10 °C/min to 150 °C, hold at 150 °C for 3 min, heat at 3 °C/min to 210 °C, and heat at 30 °C/min to 350 °C. Recording of the FID began when the GC reached 150 °C.

### DFRC Analysis

The DFRC analysis was performed on 20 mg samples of biomass according to published protocols.^41^ Bisphenol E was used as an internal standard.

### HSQC NMR Studies

The HSQC studies were performed using the standard method previously described.^42,43^ Herein, extracted biomass (1.0 g) was ball-milled for 4 h (interval: 6 min; break: 6 min; 40 cycles) using a Fritsch (Idar-Oberstein, Germany) Pulverisette 7 Mill with zirconium dioxide (ZrO_2_) vessels (50 mL) containing ZrO_2_ ball bearings (10 mm × 10) spinning at 600 rpm. Ball-milled biomass (50 mg) was then dissolved/swollen in DMSO-*d*_6_/pyridine-*d*_5_ (0.6 mL, 4:1 v/v) in a 5 mm external diameter NMR tube under sonication. HSQC spectra were acquired on a Bruker Biospin (Billerica, MA, USA) NEO 700 MHz spectrometer equipped with a 5 mm quadruple-resonance ^1^H/^31^P/^13^C/^15^N QCI gradient cryoprobe with inverse geometry (proton coils closest to the sample). Bruker’s Topspin 4.2 software (MacOS) was used to process spectra. The central solvent peaks were used as internal references (δ_C_/δ_H_ DMSO-*d*_6_ 39.5/2.49 ppm).

### Saponification

Saponification was performed according to a published method.^44^ Briefly, biomass (20 mg) was dispersed into NaOH solution (2 M, 2 mL) under sonication for 30 min. The suspension was treated for 20 h at room temperature (21 °C). 4-Hydroxy-3-methoxybenzoic acid (vanillic acid, 0.112 mg) was added as an internal standard. The solution was acidified with 1 M HCl solution (7.5 mL) and extracted into dichloromethane (3 × 10 mL). Extracts were combined, evaporated, silylated, and analyzed by GC-FID. Quantification used response factors derived from independent calibration curves for each authentic compound vs the internal standard.

## Results and Discussion

A summary of the most important hydrogenolytic monomer yield data, along with lignin contents of the various lines and the yields of phenolic acids (PAs) from hydrogenolysis vs saponification, is presented in Table 1; calculations of the syringyl/guaiacyl (S/G) ratios and their measurement from the DFRC method and from NMR for comparison, as well as yield data on a whole-cell-wall basis, are provided in the Supporting Information (Table S1). The Table 1 rows are delineated into the four sections described above; because of the quirks of the various biomass sources, an absolutely clean delineation of the crucial factors is not possible. We therefore discuss the high-S poplar in a section with softwoods below, for example, because both represent lignins from essentially a single monomer.

**Table 1:** Biomass Lignin Levels, and Releasable Monomer Yield and Composition

	KL^a^	∑SG^b^	∑SG*^c^	∑SG**^d^	%S^e^	S/G^f^	ΣPA^g^	ΣPA^h^	∑M^i^
method	KL	Hyd	Hyd	Hyd	Hyd	Hyd	Hyd	Sap	Hyd
basis	CW	KL	∑SG	∑SG	∑SG	mol	KL	KL	KL
unit	wt %	wt %	rel-wt %	rel-wt %	mol %	(ratio)	wt %	wt %	wt %
spruce	28.1 ± 1.1	15.5 ± 0.1	88.6 ± 2.4	92.8 ± 1.8	0.0	0.00			15.5 ± 0.1
balsa	23.3 ± 1.6	42.5 ± 1.4	80.9 ± 1.1	91.9 ± 0.8	70.4 ± 0.1	2.37 ± 0.01			42.5 ± 1.4
kenaf	10.5 ± 0.1	43.8 ± 0.5	88.5 ± 5.0	92.1 ± 4.4	69.4 ± 2.1	2.28 ± 0.23			43.8 ± 0.5
maple	21.9 ± 0.8	44.0 ± 4.1	90.8 ± 3.2	93.9 ± 2.4	69.8 ± 0.1	2.31 ± 0.01			44.0 ± 4.1
walnut	23.2 ± 0.3	45.1 ± 1.6	85.6 ± 0.6	89.7 ± 0.6	68.8 ± 0.4	2.20 ± 0.04			45.1 ± 1.6
oak	25.7 ± 1.0	43.4 ± 0.7	85.9 ± 0.2	90.9 ± 0.2	71.5 ± 0.3	2.50 ± 0.03			43.4 ± 0.7
beech	21.7 ± 0.7	46.4 ± 2.1	84.6 ± 0.2	89.4 ± 0.1	70.7 ± 0.2	2.41 ± 0.03			46.4 ± 2.1
birch	19.6 ± 0.8	48.2 ± 0.1	86.3 ± 0.1	90.2 ± 0.1	78.8 ± 0.4	3.72 ± 0.10			48.2 ± 0.1
willow	21.2 ± 1.6	51.7 ± 0.1	86.9 ± 1.7	91.1 ± 1.5	73.9 ± 0.7	2.83 ± 0.10	1.21 ± 0.09	1.09 ± 0.12	52.9 ± 0.9
aspen	16.9 ± 0.4	53.6 ± 0.1	86.2 ± 0.3	91.4 ± 0.3	75.0 ± 0.3	3.00 ± 0.04	1.17 ± 0.04	0.98 ± 0.04	54.8 ± 0.1
poplar	20.0 ± 0.3	47.7 ± 1.1	80.8 ± 0.4	89.5 ± 0.4	63.0 ± 0.1	1.70 ± 0.01	4.76 ± 0.01	3.94 ± 0.01	52.5 ± 1.1
hi-S poplar	16.7 ± 1.5	73.2 ± 0.1	85.0 ± 0.1	89.6 ± 0.1	96.0 ± 0.1	23.7 ± 0.3	0.75 ± 0.18		73.9 ± 0.1
palm EFB	20.8 ± 1.3	30.4 ± 0.2	67.8 ± 0.5	85.5 ± 0.6	68.2 ± 0.1	2.15 ± 0.13	10.04 ± 0.01	6.45 ± 0.02	40.4 ± 0.3
switchgrass	18.8 ± 0.3	19.1 ± 0.1	78.2 ± 0.4	87.3 ± 0.4	39.8 ± 0.1	0.66 ± 0.01	10.57 ± 0.01	5.40 ± 0.01	29.6 ± 0.1
corn stover	14.6 ± 0.1	14.5 ± 0.2	54.0 ± 0.9	86.3 ± 0.8	53.3 ± 0.1	1.14 ± 0.06	15.30 ± 0.01	10.45 ± 0.01	29.8 ± 0.4
wheat straw	17.6 ± 1.0	22.6 ± 0.6	75.5 ± 1.6	84.3 ± 1.5	50.2 ± 0.1	1.01 ± 0.05	7.24 ± 0.01	2.62 ± 0.05	29.9 ± 0.7

a Klason lignin (KL) content as measured, without attempts to correct for phenolic acids (PAs), on a wt % cell wall (CW) basis.

b %∑SG: percent yield from hydrogenolysis of lignin-derived S and G lignin monomers on a KL basis, i.e., % of the lignin released as monomers.

c %∑SG* (S-PrOH + G-PrOH only) on a wt % ∑SG basis, i.e., the % of total monomers that are the major arylpropanol (PrOH) monomers from hydrogenolysis.

d %∑SG** (S-PrOH + SPr + G-PrOH + G-Pr) on a wt % ∑SG basis, i.e., the % of total monomers that are the major arylpropyl (C6-C3, PrOH + Pr) monomers from hydrogenolysis.

e %S: relative S content from hydrogenolysis on a mol % S + G (∑SG) basis (%S + %G = 100%).

f Molar syringyl to guaiacyl ratio (S/G) from hydrogenolysis.

g Total phenolic acid (∑PA) content determined by hydrogenolysis; products arising from the phenolic esters in the biomass on a wt % KL basis.

h Total phenolic acid (∑PA) content determined by saponification of *p*-hydroxybenzoate, *p*-coumarate, and ferulate esters on a wt % KL basis.

i Percent yield from hydrogenolysis of all phenolic monomers ∑M, including the normal S and G lignin monomers and all PA, on a wt % KL basis.

Before discussing the features of each class, a few general points are in order. First, lignin levels in each biomass were measured on extractive-free biomass (essentially the cell wall, CW) by the Klason lignin method^40,45^ because it is considered to be the most accurate.^3,46^ We did not measure acid-soluble lignins that are sometimes used to apply a usually small correction to the total lignin value^40^ partly because the method is less valid for some types of biomass. Unfortunately, even today, the components contributing to the lignin levels measured remain poorly understood. Perhaps the CASA method,^47^ a new method for determining total biomass phenolics, may eventually allow for a better delineation of what is being measured. Second, we have simply ignored *p*-hydroxyphenyl (H) lignin components that are usually minor; we did not detect notable levels by any method (and do report estimates from NMR). Softwoods have only traces of H units in their clearwood, although levels can reach up to 30% in severe compression-wood zones.^48^ Hardwoods have typically <2% levels. Grasses are often reported as having higher values but are typically <5% H; values reported to be as high as 30% or more in some literature arise from erroneously ascribing nonmethoxylated phenolics from some analytical methods to H-units when they originate not from *p*-coumaryl-alcohol-derived units in the core lignin but from pendent *p*-coumarate units acylating arabinoxylan polysaccharides and lignins.^8^ Third and perhaps most importantly, we note that many reports in the hydrogenolysis literature do not attempt to distinguish phenolic monomers from lignin vs those from such pendent groups.^3^ In some ways, this is valid as, especially in chemical engineering and biomass processing plant applications, these compounds are indeed phenolic monomers produced by hydrogenolysis of the biomass. However, it is important here to clearly delineate those phenolic monomers deriving from the core lignin (and therefore involving real depolymerization reactions) from those deriving from such pendent groups. This distinction is particularly noteworthy because these components simply acylate the biomass and can therefore be readily “clipped off” by hydrolysis reactions that are much milder than those required to cleave ethers.^2,7,37,38^ Because of the ability to derive valuable commodity chemicals from these esters (or the acids released from them), research into elevating their levels on the plant cell wall is ongoing.^2,3,49−56^ These phenolates arise from biomass sources that derive their lignins, in part, from monolignol conjugates, such as the monolignol *p*-coumarates (ML-*p*CA) in grasses and monolignol *p*-hydroxybenzoates (ML-*p*HB) in the separately delineated hardwoods in Table 1 from willow (*Salix*), poplar and aspen (*Populus*), and palm (Arecaceae).^7^ In a more basic sense, it allows us to more clearly define how the core lignins behave in these classes of biomass, how important the phenolic monomers from these pendent groups may be, and how they might affect the distribution of phenolic monomers in the product mix (because of the competing hydrolysis and hydrogenolytic pathways, as illustrated in Figure 1). Because the hydrogenolytic products from lignins vs clip-offs are readily distinguished, we can also note that hydrogenolysis may provide a single-method alternative for measuring these esterified phenolic acids, usually measured by separate saponification, along with the determination of the S:G(:H) nature of biomass, typically measured by analytical thioacidolysis,^57^ the DFRC (derivatization followed by reductive cleavage) method,^41^ or NMR.^42^

Although we will discuss the biomass groups under the headings above, it became convenient to group the figures slightly differently. For example, it made sense to showcase the results from the most homogeneous lignins, the softwood (G-lignin) and the high-S poplar (S lignin), in the same figure (Figure 2).

### Gymnosperms/Softwoods

Softwoods contain essentially G-only lignins that have no associated phenolic acid conjugates (Figure 2A). In that sense, they are clean, having only one type of aromatic unit. A simpler array of monomeric products is therefore produced from any of the degradative depolymerization methods, including hydrogenolysis/RCF. We chose only a single softwood for the simple reason that studies over many decades have shown the lignins to be incredibly structurally similar, even back to the ancient gymnosperm *Ginkgo*.^58^ In fact, guaiacyl lignins produced in angiosperms by knocking out the hydroxylase [ferulate 5-hydroxylase (F5H)] crucial for syringyl lignin production are also similar.^54^ However, lignins derived from a single monolignol remain complex, with no defined sequences of units because of the combinatorial radical coupling nature of lignification, and the number of isomers possible for any oligolignol is astronomical because the coupling reactions are completely racemic.^8,59^ The distribution of interunit linkage types is near-invariant, however, attesting to the similar endwise-coupling process of *in planta* lignification that is almost certainly radical-limited and controlled by diffusion and slow monomer supply.^60^

The hydrogenolytic monomer yield from spruce is low (15.5% on a Klason lignin basis, Table 1). Guaiacyl (G) lignins are more “condensed” (having more monomer units connected by C–C bonds, units **B**–**E**, Figure 2) primarily because of the available 5-position on the monomer (coniferyl alcohol) and on phenolic-G end-groups on the polymer. More strictly, and because of this, there is a lower level of units flanked by two β-ethers, i.e., monomeric units that are both β–O–4- and 4–O−β-linked, the only units in the polymer (apart from β-ether end-units in which only a single β-ether is involved) that can release a monomer. As noted, all hydrogenolytic monomers are G monomers (Figure 2D), and because of the Pd/C catalyst used here, nearly 93% of those monomers have a three-carbon (propyl) sidechain (the ∑SG** column in Table 1); in fact, fully 88.6% of the monomers are the single compound guaiacylpropanol (G-PrOH, the ∑SG* column in Table 1). The yield deficit (as compared to hardwoods below) is therefore offset by the simplicity of the product distribution and the relative purity of the major monomer.

### Angiosperms/Hardwoods/Dicots

**Figure 3:**
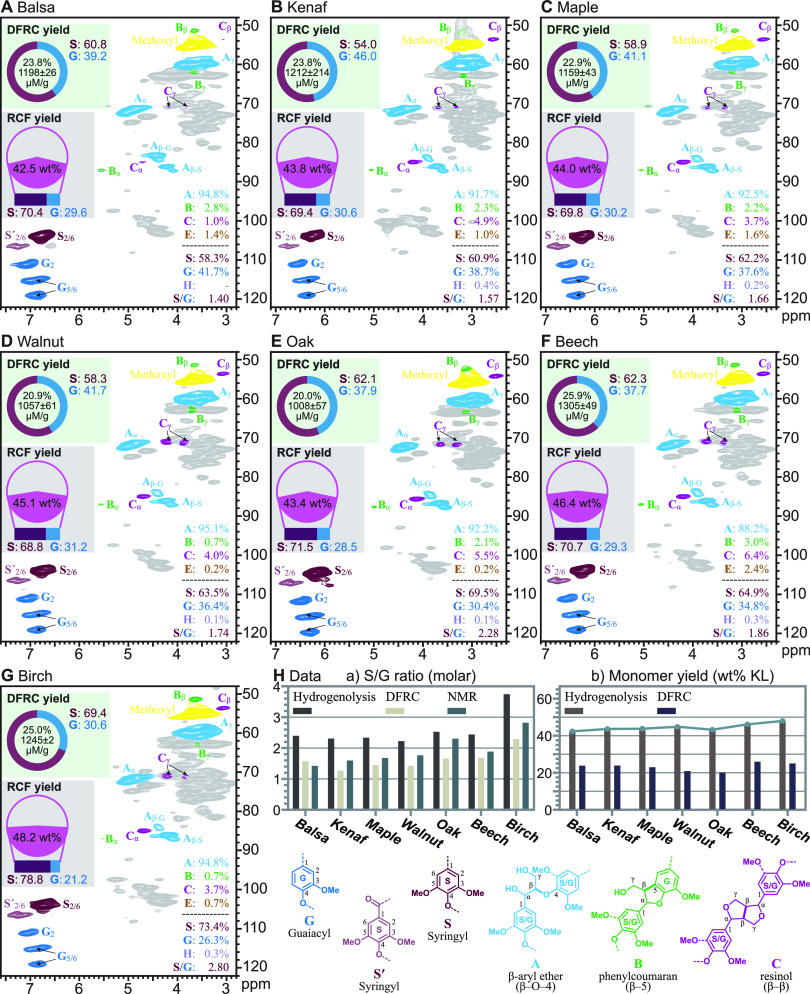
HSQC NMR spectra along with hydrogenolytic and DFRC monomer yields from seven hardwoods/dicots with normal SG lignins and no phenolate pendent groups. (A–G) NMR spectra, DFRC data (wt % of KL and mol % S and G monomers in each case), and analogous hydrogenolytic data for the seven samples in this category. (H) The S/G ratios and hydrogenolytic monomer yields from the lignins in this range of hardwoods. (a) The S/G ratio (mol %) as determined via hydrogenolysis, via the ether-cleaving DFRC method, and via HSQC NMR volume integration. (b) Phenolic monomer yields (wt % KL) from hydrogenolysis vs from analytical DFRC.

Hardwoods are syringyl-guaiacyl (SG) lignins. The syringyl monomer (sinapyl alcohol) and syringyl units in the polymer do not have the 5-position available for radical coupling and therefore produce less-condensed lignins. In fact, the propensity of sinapyl alcohol to form β–β-coupled dimers (syringaresinol, a condensed unit) at the start of lignification is higher than for coniferyl alcohol, but there is only one such unit per linear portion of the chain,^59^ so the β-ether levels are higher in hardwoods than in softwoods. In retrospect, the range in S/G ratios of the samples chosen was not huge (Figure 3), although the birch sample (with a higher S/G) is sufficient to make some inferences. In addition to measuring the S/G from the hydrogenolytic monomeric products, we also measured S/G ratios by our DFRC method and by NMR (Figure 3, Table S1). The values differ and do not even quite track linearly, but this is because each method measures different entities. Specifically, NMR measures, in principle, the S/G of the entire lignin fraction,^42^ whereas DFRC measures the S/G only of the monomers released by cleaving β-ethers;^41^ in principle, hydrogenolysis measures monomers released by cleaving those same β-ethers but also has some contribution from cleaving biphenyl ethers (5–O–4-linked units).^14^ Also, although DFRC provides a convenient method for cleaving such ethers, the reaction is not quantitative, and for example, thioacidolysis typically gives 10–20% higher levels of monomers, but the S/G values of the two methods closely agree;^61^ a complication that has largely proven useful in research into the incorporation of monolignol conjugates into lignins is that γ-esters remain completely intact after DFRC, whereas they are partially cleaved under thioacidolysis.^62,63^ The NMR values perhaps track better with the hydrogenolytic data (Table S1) but are lower except for those from the exceptionally high-S poplar lignin. NMR is perhaps expected to give slightly lower S/G ratios because the lignin that remains unconverted to monomers is likely more condensed and more G-rich. More quantitative NMR methods continue to be explored for lignins.^21^ There simply is no independent absolute method, so there is no basis for declaring one method to be superior or more accurate. The values are reported in Table S1 just for comparison, and we focus here on the levels released by hydrogenolysis.

This set of biomass materials is grouped because the lignins are not derived from significant levels of monolignol conjugates; i.e., they have essentially no phenolic acid (PA) levels to report. Some hardwood lignins, however, may be derived from unknown but usually low levels of monolignol acetate (ML-Ac) conjugates. Kenaf has been found to have a high-syringyl lignin that is particularly high in ML-Ac conjugates, but this is only in the kenaf bast fiber;^64^ here, we chose the core that has a more normal lignin that lacks significant conjugate incorporation.

The hydrogenolytic monomer yields were rather consistent (42–48%, as in the ∑SG column in Table 1), with the birch lignin having the highest yield presumably because of its higher S/G. As noted previously, the monomer yields from hydrogenolysis (RCF) are among the highest of any of the degradative methods. The total arylpropanoid yields (∑SG**, Table 1) were within a narrow range (89–94% of the total monomers, ∑SG) and about the same as for the softwood (93%) on a lignin basis. The monomer mix, however, consists of S and G monomers and so is logically more complex. The arylpropanol (∑SG*, S-PrOH + G-PrOH) yields were a respectable 81–91% of the total monomers. We comment more on factors that affect the differences between these two values below.

### Hardwoods Incorporating ML-*p*HB Conjugates

Next is a set of hardwoods that are different because they have lignins derived in part from monolignol *p*-hydroxybenzoate (ML-*p*HB) conjugates; i.e., they are lignins in which some of their γ-OH groups are acylated by pendent *p*-hydroxybenzoates.^7,27,65,66^*p*-Hydroxybenzoic acid (*p*HBA) is the first of the so-called “clip-offs” that can be readily released to produce a mildly valuable and simple product stream in the biorefinery; *p*-hydroxybenzoates have myriad uses from parabens^67^ to precursors for commodity chemicals such as *p*-aminophenol and pharmaceuticals such as acetaminophen (or Tylenol in the United States).^68^

*Populus* and *Salix* genera and the Arecaceae (palm) family are important because they are commercial species with bioenergy value. Poplar and willow can be coppiced and therefore grown as a crop plant.^69^ Oil palm proliferation is not to be encouraged but is already in production, and the waste products from the industry are underutilized.^27,66,69,70^ This set of plants has a reasonable range of S/G and varying *p*HB levels. The palm sample is different in that it is the empty fruit bunches (EFBs),^27^ not the wood, and has a high level of *p*HB; it is therefore treated slightly separately. The high-S poplar is a transgenic with unprecedentedly high syringyl lignin content and is also described separately below.

**Figure 4:**
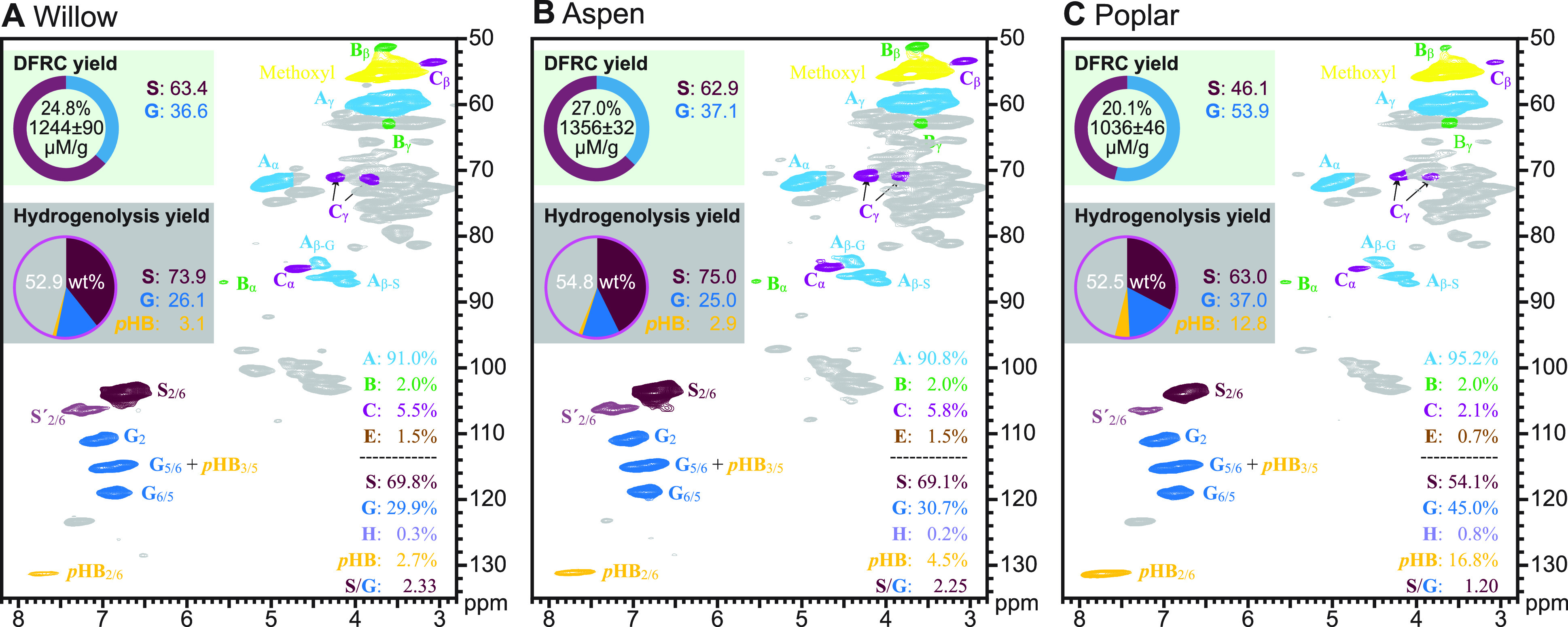
HSQC NMR spectra along with hydrogenolytic monomer yields (wt % of KL) and DFRC monomer yields (wt % of KL, but lacking contribution from ML-*p*HB conjugates), mol % S and G monomers in each case, and *p*HB ratios (with mol % *p*HB on an S + G = 100% basis) from hydrogenolysis from three hardwoods with naturally *p*-hydroxybenzoylated lignins derived from lignification incorporating ML-*p*HB conjugates. (A) Willow. (B) Aspen. (C) Poplar.

Hydrogenolytic yields from the willow, aspen, and poplar were again high and fairly consistent (∑M 52.5–55% and ∑SG 48–54%, Table 1 and Figure 4). The total arylpropanoid yields (∑SG**) were, as for the generic hardwoods, high and within a narrow range (89.5–91.5%) on a lignin basis; the arylpropanol (∑SG*, S-PrOH + G-PrOH) yields were, however, lower at 81–87%. The larger difference between the total arylpropanoids and just the arylpropanols (∑SG** – ∑SG*) is logically related to the conjugates, as noted in Figure 1. The explanation is simple: acylated β-ether units undergo hydrogenolysis to produce the arylpropanes even more readily than their unacylated analogs (Figure 1). This effect is more strikingly seen in the highly acylated lignins from palm EFB and the grasses as described below. This effect alone justifies the special consideration given to lignin acylation here. Although the DFRC monomer yields track moderately well with hydrogenolysis yields for the softwood and normal hardwoods (and with the grasses, but with significantly lower yields as explained below in the Monocots/Grasses section), the levels reported for this special series track less well (Table S1). This is because we were unable to measure the levels of ML-*p*HB conjugates by DFRC; they failed to survive the GC conditions. The poor tracking comes from the different *p*HB levels in the various samples and particularly underquantifies the syringyl units (ΣS, DFRC) because such *p*HBs predominantly (∼90%) acylate syringyl units.^27^ The palm EFB sample, because of its high *p*HB levels, appears particularly discrepant because the ML-*p*BA conjugates could not be quantified here [see the note at the bottom of Table S1].

Two samples in this section are special and deserve independent comment. First, the incredibly high-S poplar transgenic releases, as has been noted before,^35,71^ particularly high yields of monomeric phenolic products, 73.9% here (Table 1). This is logical because high-S lignins have two notable features: they are essentially uncondensed and linear, and they have high β-ether levels (because the 5-coupling pathways to condensed units are unavailable).^29,30,34,35,72^ Another advantage is that, like the G-only lignins from softwoods, the effectively S-only lignin in this transgenic, as seen in the NMR (Figure 2B), releases a monomer mix that is considerably simpler, being composed of essentially only S monomers. We have often noted the particular value of such high-S lignins, and it should be obvious that if the monomeric products, syringylpropanoids, were valuable to access, these transgenics would be particularly attractive for their enhanced pulping properties^29^ and their ability to deliver high yields of a simple mix of aromatic monomers from their lignins.^35,71^ Here, the level of syringylpropanoids (∑SG**, which is essentially just ∑S** = S-PrOH + S-Pr) is similarly high as that from the wild-type poplar (∼90%), and the syringylpropanol (∑SG*, which is essentially just ∑S* = S-PrOH) proportion is again 85%; i.e., hydrogenolysis of this special material produces a 74% yield of monomers, some 90% of which are syringylpropanoids and 85% of which is a single compound, syringylpropanol. Not long ago, the notion of obtaining such yields and purity of products from lignins would have been unimaginable.

**Figure 5:**
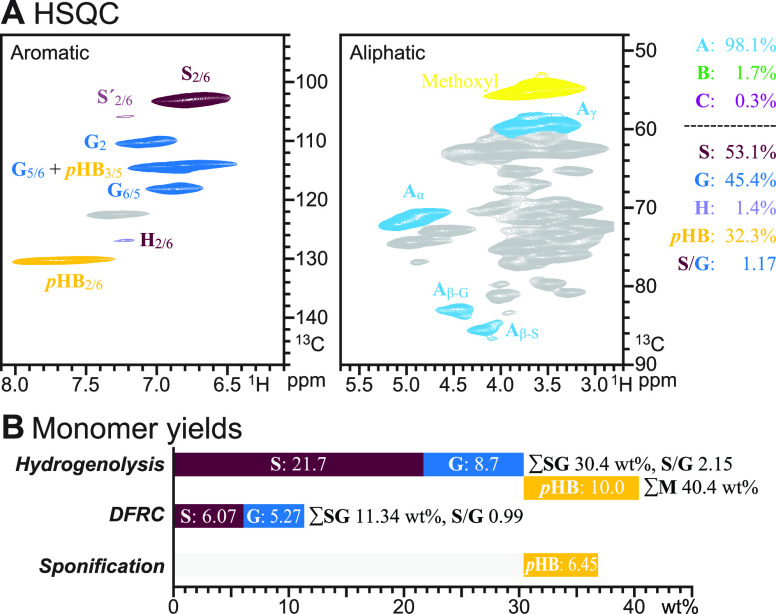
HSQC NMR, along with hydrogenolytic, DFRC, and saponification monomer yields (wt % of KL), from oil palm empty fruit bunch residual biomass that has a naturally *p*-hydroxybenzoylated lignin derived from lignification incorporating ML-*p*HB conjugates. (A) Aromatic and oxygenated-aliphatic regions of the HSQC NMR spectrum. (B) Phenolic monomer yields (wt % KL) and S/G (molar) data via hydrogenolysis (RCF), DFRC (but lacking contribution from ML-*p*HB conjugates), and simple saponification aimed at releasing the *p*-hydroxybenzoic acid from the pendent *p*-hydroxybenzoate esters.

As the second special material, the oil palm EFB results are considerably different (Table 1, Figure 5). Again, this is in part because this sample is from the empty fruit bunches following oil-seed removal and is not from the wood. That product is high in lignin but has a high *p*HB content, 10% (on a lignin basis) from our hydrogenolysis here and 6.5% from saponification (Table S1); from prior studies, acylation by *p*HBA is known to be essentially all on syringyl units.^27^ The yield of hydrogenolytic monomers from the core lignin, ∑SG = 30.4%, was lower than for any of the hardwood samples. It is not immediately obvious why this is the case given the standard S/G, the high *p*HB content notwithstanding, but the latter effect was also noted with the grasses, as discussed below. The relative level of arylpropanoids (∑SG**) was a respectable 86%, in line with that from the other hardwoods. The significantly lower arylpropanol level (∑SG*, 68%) is clearly attributable to the level of conjugates in this sample, conjugates that again more readily undergo hydrogenolysis to the arylpropane level (Figure 1).

The total level of phenolic monomers, as usually reported for such samples, is included in the last column (∑M) of Table 1. The *p*HB groups on lignins are also released by hydrogenolysis to produce *p*HBA (or its methyl ester) essentially quantitatively. Only a small augmentation in the level of monomers is seen to result from these units with poplar, aspen, and willow woods, but the palm EFB sample that has some 10 wt % of its lignin as *p*HB units delivers a total monomer yield (40.4%) that is much higher than the yield of core-lignin monomers (30.4%). This feature will be noted again below in the grasses that have similar levels of PAs in the form of *p*-hydroxycinnamates and reinforces our contention that the monomers from these clip-offs should be tallied separately from the true core-lignin-derived monomers.

### Monocots/Grasses

**Figure 6:**
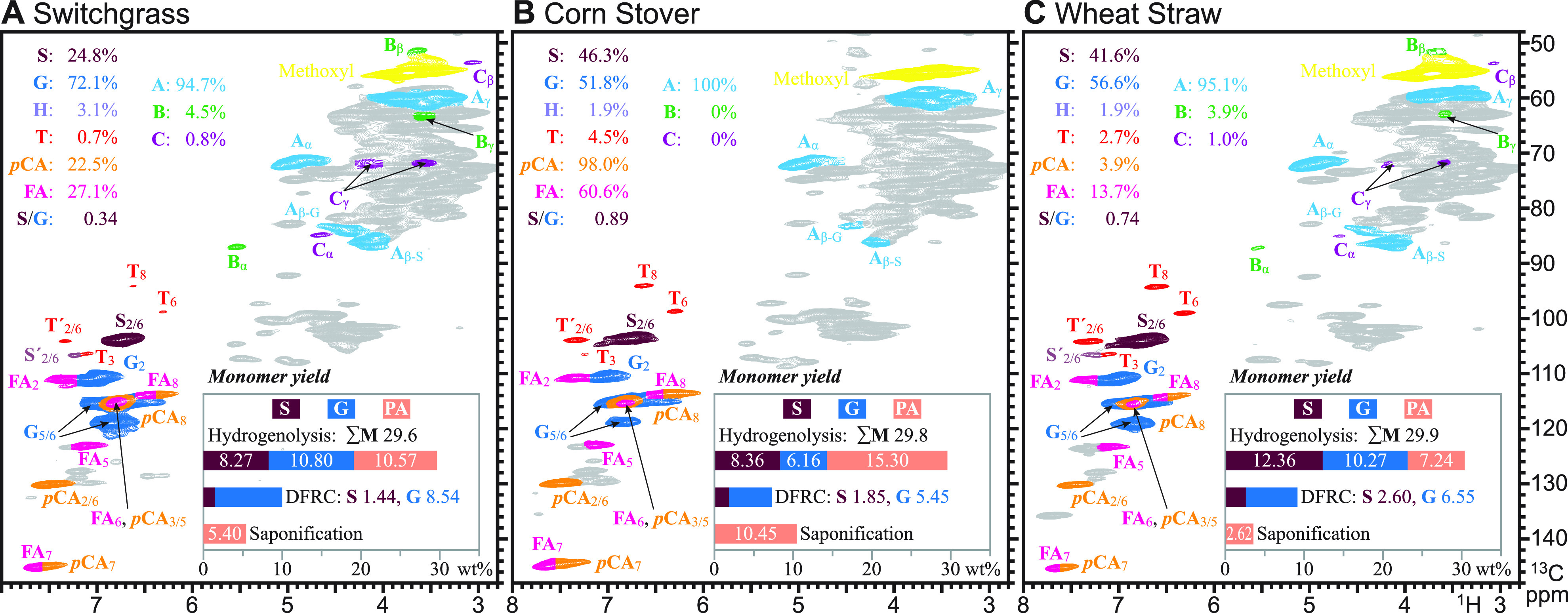
HSQC NMR, along with hydrogenolytic, DFRC, and saponification monomer yields (wt % of KL), from three grasses (monocots) that have naturally *p*-coumaroylated lignins derived from lignification incorporating ML-*p*CA conjugates and arabinoxylan hemicelluloses that are naturally acylated with both *p*-coumarate (*p*CA) and ferulate (FA). (A) Switchgrass. (B) Corn stover. (C) Wheat straw. PA signifies the total phenolic acid levels, in this case, *p*CA and FA (or their methyl esters).

The last class of samples is the grasses, represented by switchgrass, maize/corn, and wheat (Table 1, Figure 6). Grasses are long-known to have *p*-coumarate pendent groups on their cell walls, including on lignins.^7,73−75^ They also have high levels of ferulates and *p*-coumarates acylating arabinoxylan polysaccharides.^7,74,76^ Ferulates are also now known to acylate monolignols and thereby incorporate into lignins in grasses and a few other plant lines.^24^ If the phenolic acids are included in the monomer yields (ΣM, Table 1), the yields are modest (29.6–29.9%) but substantially lower than for the set of hardwoods. The total yields of hydrogenolytic monomers from just the core lignin (ΣSG, Table 1) are particularly low, 14.5–22.6%. These lignins certainly have lower S/G values than the hardwoods but still have a sufficient S/G to anticipate higher β-ether levels than the softwoods, for example.

The low yield (on a lignin basis) of hydrogenolytic monomers from grasses has always been a mystery; we offer some thoughts from the literature on grasses and the data here. We do not think the dilemma comes from neglecting the H-unit contribution (as no/few H-unit monomers were detected in our hydrogenolytic analyses) but do recommend further study. There are other features of grass cell walls and lignins that likely contribute, however. First, it has long been known that grass arabinoxylans are acylated by both *p*-coumarate and ferulate.^7,74^ Ferulate, in particular, has been well implicated in extensive cell wall cross-linking, both polysaccharide–polysaccharide cross-linking via ferulate (dehydro)dimerization (and higher oligomerization)^7,44,77^ and, importantly here, lignin–polysaccharide cross-linking; both processes have been shown to be via radical coupling.^7,78^ Ferulates on arabinoxylans may act as initiation/nucleation sites for lignification.^7,79^ It has also been well established that ferulates are involved in even more cross-coupling pathways than the monolignols themselves; for example, whereas the coniferyl alcohol monomer does not undergo 4–O–5 or 5–5 coupling, ferulates do.^44,80^ The array of possible ferulate coupling and cross-coupling products is illustrated in Figure S2 of a paper on the introduction of monolignol ferulates into lignification.^23^ Grabber has estimated, from model studies and from studies on isolated cell walls, that only 40% of ferulates incorporated into lignins can be recovered following hydrolysis of ethers at high temperature.^81^ It is likely that hydrogenolysis has fewer limitations and should be able to release ferulate-derived monomers from ferulate-8–O–4-ethers, for example, that cannot, despite being β-ethers, be released in good yield upon high-temperature base treatment^81^ or with methods such as DFRC without untested modifications because ferulate’s ubiquitous esters are not cleaved and monomers are therefore not released.^63^

Another feature leading to lignin cross-linking in grasses has only more recently come to light. We spent some 15 years attempting to develop a novel approach to introduce readily cleavable bonds in the lignin backbone. That method of introducing novel monolignol ferulate (ML-FA) conjugates into lignification was shown to be possible by providing *Arabidopsis* and poplar with the necessary genes to produce the enzymes to biosynthesize such conjugates.^23^ The DFRC-based methodology that was developed to document this success, allowing us to certify not only that the engineered plants were making such conjugates but that they were being incorporated into the lignins by the usual radical coupling mechanisms, produced an important revelation: poplar (but not *Arabidopsis*) was already making and incorporating low levels of ML-FA conjugates into its lignin.^23^ A subsequent phylogenetic survey showed that “all” grasses, the commelinid monocots in particular, were utilizing ML-FA conjugates in their lignification, as were some hardwoods, but not softwoods (or many other hardwoods, to our current level of detection at least).^24^ In grasses, the primary conjugate was sinapyl ferulate, whereas hardwoods favored coniferyl ferulate. This means that, in addition to the ferulates on arabinoxylans, grasses had another mechanism for incorporating ferulate into their lignins and, as noted, ferulates may be involved in extensive cross-coupling reactions.^7,59^ The myriad ways by which ferulate can enter lignification, many of which result in non-hydrogenolysis-cleavable products, have been diagrammed in Figure S2 of the monolignol ferulate paper.^23^ We suggest here that the reason why grasses release significantly lower levels of lignin-derived hydrogenolytic monomers (∑SG, Table 1) than hardwoods with comparable S/G ratios is largely because of the level of ferulate incorporation, from the two sources noted, into lignification. As partial evidence, we note that the fraction of the total PAs documented in Table 1 that is ferulate (vs *p*-coumarate) is 48.5–63 wt % (Table S1). Because ferulates can only be partially recovered by any of the degradative methods,^23^ the actual levels of ferulates incorporated will exceed those of *p*-coumarates that can, in principle, be fully recovered because they are essentially all simple ester-linked pendent groups.

An obvious approach to enhancing monomer yields (if PA monomers are included in those yields), or simply of the PAs available to be clipped off,^37,38^ is to select, breed, or engineer biomass with increased levels of PAs in their cell walls, either/both on their lignins or/and on their polysaccharides. Examples of the latter include an examination of strategies to enhance *p*CA levels on lignin in the grass model *Brachypodium*(53) and in maize,^82^ engineering ML-*p*CA biosynthesis into poplar,^51,83^ and elevating the *p*HB levels in poplar.^84^ If the reasoning in the paragraph above is valid, an approach, not being considered to our knowledge, to elevating the levels of monomers derivable from the core-lignin structure in grasses would be to repress the incorporation of ferulate esters into lignin. This requires two biochemical processes to be knocked out: the acylation of lignin monomers by ferulate by now-known genes for the feruloyl-CoA:monolignol transferase (FMT)^23,24,53−55^ and the suppression of arabinoxylan feruloylation, genes that have been speculated but not entirely validated.^49,85^ As the latter process is crucial for cell wall (polysaccharide–polysaccharide and polysaccharide–lignin) cross-linking in grasses and as such feruloylation of polysaccharides has not been successfully eliminated, we strictly do not know the agronomic impact of knocking out such pathways; given that other plant lineages function well without such cell wall cross-linking mechanisms, however, we assume that the knockouts would at least not be lethal.

### PA Quantification by Hydrogenolysis vs Saponification

In addition to producing among the highest phenolic monomer yields, hydrogenolysis appears to warrant further exploration as a phenolic acid measure. The yields of PAs from hydrogenolysis reported here are invariably higher than those from saponification (Table 1). We are rather confident of the hydrogenolytic yields, as quantification used calibration curves and an internal standard. There are indications that saponification may need optimizing for the various sample types, but we used conditions well-documented over decades for their analytical purposes. Neither was the hydrogenolysis optimized; we used conditions determined in previous studies by ourselves and others to be optimal for lignin monomer yields.^14,16−18,86^ Given that the highest yields produced among valid methods are optimal, it is tempting to suggest that hydrogenolysis may be the best method for quantifying phenolates in biomass. Access to the entirety of the lignin macromolecular complex in the cell wall is a well-known problem in analytical methods that do not involve true solubilization. It seems reasonable to conclude, both from the high lignin monomer yields and from these high PA yields, that access is mitigated in the hydrogenolytic system. Perhaps no data are more telling than the enormously higher yield of PA from hydrogenolysis vs saponification in the case of wheat straw. From the details of the analysis (Table 1 and Table S1), this material has a higher ferulate proportion (63 wt %) than the other grasses (48.5–53 wt % of the PA, by weight, by hydrogenolysis) but tellingly much lower by saponification (35 wt %). The poor ferulate release by saponification suggests an accessibility problem that may or may not be remedied by optimization of the treatment.

As alluded to above, there are other possible reasons for the higher PA yields, particularly for ferulate units. It seems logical that hydrogenolysis would cleave units that have been shown not to cleave via saponification. This includes 8–O–4-diferulates (on polysaccharides or incorporated into lignins) and the array of ferulate-8–O–4-S/G units derived from ferulate’s cross-coupling into lignin,^81^ as well as from the (admittedly low levels of) 4–O–5-linked units.^16,80^

We are not claiming here that hydrogenolysis is a better method for PA quantification in biomass, and it may be hard to develop into a sufficiently high-throughput operation for some labs, but researchers having the required reactors may find it to be a particularly convenient method. As alluded to in a lignin-first guidelines paper,^3^ we simply demonstrate here that hydrogenolysis is at least as good as other methods without optimization; i.e., if researchers have a reactor, it is a convenient way to obtain good yield data for the various PAs and has the advantage that no changes are needed to the method used to quantify lignin monomer production; both determinations, with “any” required level of detail on individual components, are accomplished in the same experiment.

### Impact of Lignin Composition on Hydrogenolytic Monomer Yields

**Figure 7:**
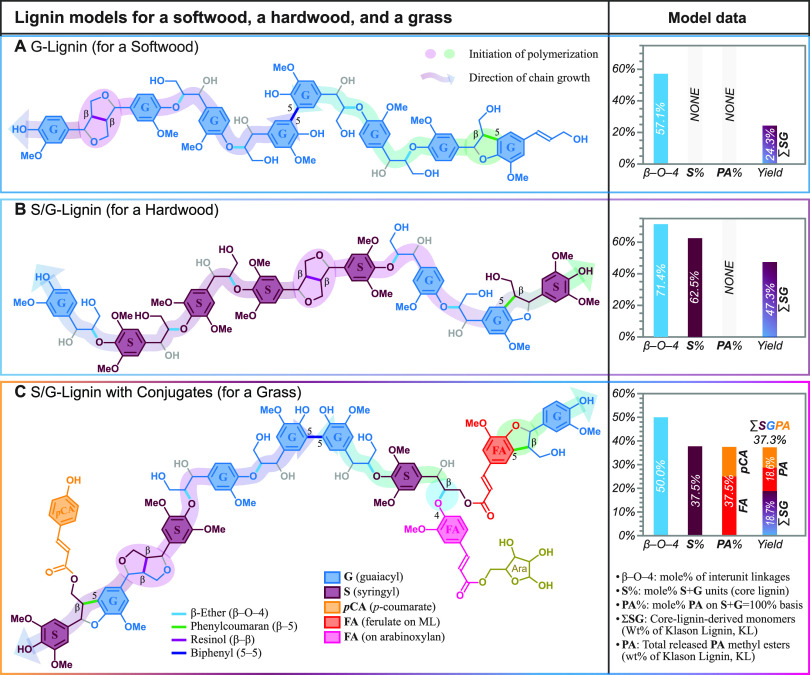
Models illustrating the impact of the composition of various lignin classes from monolignols and their conjugates on hydrogenolytic monomer yields. (A) Softwood. (B) Hardwood. (C) Grass. The lignin model for each class is simply a plausible model constructed according to the free-radical coupling theory of lignification, but the comparison shows how structural variations and the incorporation of hydroxycinnamate esters can affect the quantification of various units in the core lignin, the release of hydroxycinnamates, and the yields of hydrogenolytic monomers. See the explanation in the main text.

To illustrate the impact of lignins’ composition from monolignols and their conjugates on hydrogenolytic monomer yields, plausible lignin models for each class were constructed here in ChemDraw according to the free-radical coupling theory of lignification (Figure 7). These models show how structural variations and the incorporation of hydroxycinnamate esters can affect the quantification of various units in the core lignin, the release of hydroxycinnamates, and the yields of hydrogenolytic monomers.

Key features of the models and their cleavage to monomers include the following. Eight monolignols were used to build the core-lignin chain for each because this is sufficient to produce lignins with reasonable β-ether contents, %S (or S/G ratio), and phenolate content and produces theoretical monomer yields comparable to those seen here from hydrogenolytic depolymerization results. Four well-identified lignin interunit linkages, β–O–4, β–5, β–β and 5–5, were used to link lignin units together. The β-ether, with its β–O–4 linkage, is the lignin motif involved in depolymerization; β–β, β–5, and 5–5 linkages represent C–C bonds with the β–5 and 5–5 units representing major differences between G and S units (and therefore softwood lignins from the others) and with the 5–5 units indicating the coupling of two oligomeric or polymeric chains.^6,59,87^ The yields are calculated assuming that all the ether and ester bonds are cleaved whereas the C–C bonds remain intact; arylpropanols are assumed here to be the only product of the hydrogenolytic cleavage of β-ether units because they are the predominant product (∼90%) produced using Pd/C (whereas arylpropanes are major with Ru/C),^88,89^ and the methyl hydroxycinnamates result from ester cleavage (transesterification) only if they are not further C–C-bonded.

#### Softwood Lignin

To model the softwood’s lignin, a polymer chain (G-Lignin, Figure 7A) grows from both the β–5 (right) and β–β (left) dimeric starting units via further 4–O−β-coupling, and two growing chains couple via 5–5 bonding (middle). This produces a 1499.57 molecular weight lignin 8-mer of 100% G units in which four of the seven (57.1 mol %) interunit linkages are cleavable β-ethers (cyan bonds) but result in the release of only two guaiacylpropanol monomers and therefore in a hydrogenolytic lignin monomer yield (∑SG, but actually only ∑G in this case) of 24.3 wt % (based on the total lignin).

#### Hardwood Lignin

The hardwood’s lignin was modeled with an eight-unit polymer (the S/G-Lignin, Figure 7B) that has five sinapyl-alcohol-derived S-units and three G-units. A β–β unit from sinapyl alcohol dimerization is used to start the polymer chain (as is common in hardwoods), and the lignin contains mainly β-ether units and one phenylcoumaran via a β–5 linkage from cross-coupling of sinapyl alcohol with a G phenolic end-unit, to illustrate another uncleavable unit containing an S unit; no 5–5 polymer chain coupling is presented (as these are usually minor in hardwoods). This produces a 1667.72 molecular weight lignin 8-mer of G and S units in which five of the seven (71.4%) interunit linkages are cleavable β-ethers (cyan bonds) resulting in the release of two guaiacylpropanol and two syringylpropanol monomers and therefore in a hydrogenolytic lignin monomer yield (∑SG) of 47.3 wt % (based on the total lignin). Although these models are not perfect, this illustrates how S/G lignins typically result in lignins with higher β-ether contents and cleave to monomers in higher yields than softwoods represented in Figure 7A.

#### Grass (Monocot) Lignin

The monocot’s lignin includes the various ways in which the SG lignin can also incorporate hydroxycinnamates. To make the lignin model of monocots compatible with the others, the lignin was again generated from eight monolignols (three S and five G units) but in which two of the monolignols are hydroxycinnamate conjugates, one *p*-coumarate (*p*CA), and one ferulate (FA). All hydroxycinnamates acylate S-units (and therefore derive from sinapyl *p*-coumarate or sinapyl ferulate) as observed prevalently in monocots.^24^ In addition, one ferulate on arabinoxylan is also incorporated. As noted in monocot lignification, this ferulate may actually be a nucleation site for lignification, a site at which the first monolignols (cross-)couple.^7,79^ However, we have this coupling with another monolignol that in the next step also becomes involved in a 5–5-coupled unit (by coupling with another growing chain). Also to be noted here and somewhat analogously to the resinol dimer, these monolignol ferulate conjugates have two phenolic ends that are both capable of undergoing independent coupling events and therefore propagating the chain in two directions; here we show that a monolignol, coniferyl alcohol, undergoes β–O–4 coupling with the ferulate, but it should be recognized that this ferulate could have first undergone coupling at its sidechain 8-position with a lignin unit to produce an even more highly branched structure.^7^ The monolignol moiety in the original monolignol ferulate conjugate can undergo coupling in the same way as the canonical monolignol does; here its initial β–O–4 coupling with a ferulate on arabinoxylan is shown; the phenolic end, which remains free-phenolic after this sidechain coupling, is then free to undergo further 4–O-coupling. The other initiation in this chain is from β–β coupling to produce a resinol, as in the lignins in Figure 7A,B, here shown as a mixed G-S resinol. Again, as in the softwood, two chains come together to become 5–5-linked. A monolignol *p*-coumarate conjugate, from sinapyl *p*-coumarate as is predominant in monocots,^7,62,75^ is also coupled in (left end). The core-lignin chain is therefore composed of three S units and five G units along with two FA moieties, one deriving from a sinapyl ferulate conjugate and one from a ferulate acylating arabinoxylan chains (as is common in monocots).

All this activity produces a 2104.14 molecular weight lignin complex (after taking off the arabinosyl moiety, i.e., just the aromatic fraction), again containing eight monolignol-derived G (five) and S (three) units in which four of the eight (50.0%) interunit linkages (that include the two ferulates) are cleavable β-ethers (cyan bonds) resulting in the release of one guaiacylpropanol and one syringylpropanol monomer and therefore in a hydrogenolytic lignin monomer yield (∑SG) of just 18.7 wt % (based on the total lignin). One ether-linked ferulate and the sole pendent *p*-coumarate are also releasable, producing 18.6 wt % total phenolate (PA) monomers, for a total hydrogenolytic monomer yield of 37.3 wt %. Again, although these models are not perfect, this illustrates how, despite being an SG lignin (as in the hardwood), the incorporation of ferulates can reduce the hydrogenolytic yield of monomers derived from the core-lignin units to roughly the low levels seen from softwoods (see in Figure 7A); if the phenolic acids are incorporated into the total monomer yield, however, the total apparent yields of phenolic monomers from grasses are higher than those for softwoods that have no conjugated phenolic acids.

## Conclusions

The following points are the most notable summary observations on yields vs biomass features from the data:

1. SG-lignins produce higher hydrogenolytic monomer yields than G-lignins, for the obvious reason that the β-ether content is higher and the lignin is less condensed. The high-S lignin in a transgenic poplar produced a 74% monomer yield, 85% of which was the single monomer syringylpropanol.
2. Lignins that are homogeneous with respect to the types of aromatics they contain obviously produce the simplest product mixtures as they have only a single type of aromatic unit, G from softwoods, S from the S-rich poplar, and, as shown previously,^16,90^ catechyl units from C-lignins.
3. Total monomer yields from the lignin are not substantially different in hardwoods that derive from ML-*p*HB conjugates over the hardwoods that do not. If *p*HBA is included in the quantified monomers, then the total yields are modestly higher, mainly because of the efficiency of release of *p*HBA (or its methyl ester) from simple pendent *p*HB esters. One means of elevating total monomers yield is therefore to select or engineer biomass with high *p*HB levels; researchers are attempting to engineer such plants.^56,84^ It must be remembered, however, that this produces a different monomer that likely needs separating or should be clipped off first with an independent pretreatment. Separations of even quite complex mixtures is improving,^91^ and chemical or biological funneling can take complex mixtures to a single product or a simpler array of compounds.^4,92^
4. Grasses furnish lower yields of lignin-derived monomers than would be expected from their SG content and in comparison with hardwoods. We contend that much of this deficit is caused by ferulates, both from monolignol ferulate conjugates and from the ferulates and diferulates on arabinoxylans, all of which are involved in lignification. The monomer level improves substantially if it includes the monomers from the phenolic acids, *p*CA and FA, but it must be noted that a high proportion of these arise from their presence on polysaccharides, implying that their frequent attribution to products of lignin degradation is incorrect. Attempts to enhance *p*CA and FA levels in grasses, both on the polysaccharides and on the lignin, are under active study.^49−51,53,82−85,93^
5. Lignins derived (in part) from monolignol conjugates, likely including ML-Ac that has not been studied here, will give lower (relative) yields of the major propanol products under Pd-catalyzed hydrogenolytic conditions, possibly complicating monomer purification. It may be advantageous, both for the higher selectivity toward arylpropanol monomers and for isolating pure products, to obtain the PAs by clipping them off in a separate pretreatment, such as saponification (although we note that doing so quantitatively is nontrivial). Selecting or developing catalysts to produce desired product distributions is also a viable approach,^2,3,12,89,94^ as is engineering or selecting plants with altered PA levels and distributions.

It can be concluded that if the monomeric products have sufficient value, homogeneous lignins that have low degrees of condensation are ideal for producing the best yields of monocomponent monomers. The high-S lignin engineered in a transgenic poplar, as reported here and elsewhere, is remarkable.^35,71^ Even better yields have been reported from a C-lignin derived from caffeyl alcohol that happens to be almost a purely β–O–4-linked homopolymer;^16,90^ because the units are linked predominantly by ethers (but in benzodioxane units because of the catecholic nature of the monomers) that efficiently cleave under hydrogenolysis, 90% yields of monomers, 90% of which was a single monomer (dihydrocaffeyl alcohol), can be achieved.^16^ Such biomass, if available in quantity, would be highly desirable. Although natural C-lignins (and also 5-hydroxyguaiacyl lignins that are similarly homogeneous) exist, they are in seedcoat tissues, and it is unclear whether plants can be persuaded to produce such biorefinery-ideal homogeneous lignins in their woody biomass, but researchers are certainly exploring such possibilities.

### Note Added after Preparation of This Manuscript

Well after performing this research and collecting these data and after producing our first-draft manuscript, an interesting paper, using another catalyst, noted similar yield trends using various types of biomass (“lignin sources”)^95^ and, to some degree, assessed pendent esters vs what we are calling “core-lignin” monomers. We also became aware of work by colleagues (Beckham’s group) that is complementary (but not overlapping) and has now been published;^96^ co-publication of this and our article might have been beneficial.

## Supporting Information Available

Monomer yield and composition by various methods (Table S1) (PDF)
