# Supplementary material for: Lignin Hydrogenolysis: Phenolic Monomers from Lignin and Associated Phenolates across Plant Clades
Source: ACS Sustain Chem Eng. 2023 Jun 28;11(27):10001–17. doi: 10.1021/acssuschemeng.3c01320 (PMC10337261; doi:10.1021/acssuschemeng.3c01320)
Supplement: Supplementary file 1 — sc3c01320_si_001.pdf [file sc3c01320_si_001.pdf]

Lignin hydrogenolysis: Phenolic monomers from lignin and associated phenolates across plant clades

*Mingjie Chen,[†,#] Yanding Li, [†,¥] Fachuang Lu, [†] Jeremy S. Luterbacher, [‡] and John Ralph[†,§,]**

[†]Department of Energy, Great Lakes Bioenergy Research Center, Wisconsin Energy Institute, Madison, Wisconsin 53726, USA.

[‡]Institute of Chemical Sciences and Engineering, École Polytechnique Fédérale de Lausanne, Lausanne, Switzerland.

[§]Department of Biochemistry, University of Wisconsin-Madison, Madison, Wisconsin 53706, USA.

* Corresponding author (Email: jralph@wisc.edu).

Number of pages: 2

Number of figures: 0

Number of tables: 1

Table S1. Monomers yield and composition by various methods (Data additional to Table 1)

	**%S**[a]	**%S**[b]	**%G**[g]	**%H**[d]	**S/G**[e]	**S/G**[f]	**Yield**[a]	**G**[h]	**S**[i]	**∑SG**[j]	**∑S**[k]	**∑G**[l]	**∑S**[m]	**∑G**[n]	***p*CA**[o]	**FA**[p]	**%FA**[q]	**%FA**[r]	**∑PA**[s]	**∑PA**[t]
Method	DFRC	NMR	NMR	NMR	DFRC	NMR	DFRC	DFRC	DFRC	DFRC	Hyd	Hyd	DFRC	DFRC	Hyd	Hyd	Hyd	Sap	Hyd	NMR
Basis	∑SG	∑SG	∑SG	∑SG	mol	mol	KL	∑SG	∑SG	KL	KL	KL	KL	KL	KL	KL	KL	KL	∑SG	∑SG
Unit	mol%	mol%	mol%	mol%	(ratio)	(ratio)	µM/g	mol%	mol%	wt%	wt%	wt%	wt%	wt%	wt%	wt%	wt%	wt%	mol%	mol%
Spruce	0.0	0.0	99.1	0.9	0.00	0.00	664±70	100.0	0.0	12.0±1.3	0.0±0.0	15.5±0.1	0.0±0.0	12.0±1.3	--	--			--	--
Balsa	60.8±0.1	58.3	41.7	0.0	1.55±0.01	1.40	1198±26	39.2	60.8	23.8±0.5	31.2±1.0	11.3±0.4	15.3±0.3	8.5±0.2	--	--			--	--
Kenaf	54.0±8.4	60.9	38.7	0.4	1.25±0.41	1.57	1212±214	46.0	54.0	23.8±4.5	32.0±0.3	11.9±0.9	14.1±4.6	9.7±0.1	--	--			--	--
Maple	58.9±0.3	62.2	37.6	0.2	1.43±0.02	1.66	1159±43	41.1	58.9	22.9±0.9	32.1±3.0	11.9±1.1	14.3±0.6	8.6±0.2	--	--			--	--
Walnut	58.3±1.8	63.5	36.4	0.1	1.40±0.10	1.74	1057±61	41.7	58.3	20.9±1.3	32.4±1.0	12.6±0.6	13.0±1.1	7.9±0.1	--	--			--	--
Oak	62.1±0.1	69.5	30.4	0.1	1.64±0.01	2.28	1008±57	37.9	62.1	20.0±1.1	32.3±0.4	11.1±0.3	13.1±0.7	6.9±0.4	--	--			--	--
Beech	62.3±2.0	64.9	34.8	0.3	1.66±0.14	1.86	1305±49	37.7	62.3	25.9±1.1	34.2±1.5	12.2±0.6	17.1±1.2	8.8±0.1	--	--			--	--
Birch	69.4±0.9	73.4	26.3	0.3	2.27±0.10	2.79	1245±2	30.6	69.4	25.0±0.1	39.2±0.2	9.0±0.2	18.1±0.3	6.9±0.2	--	--			--	--
Willow	63.4±2.7	69.8	29.9	0.3	1.75±0.21	2.33	1244±90	36.6	63.4	24.8±1.9	39.7±0.3	12.0±0.5	16.6±1.9	8.1±0.1	--	--			3.1±0.03	2.7
Aspen	62.9±0.4	69.1	30.7	0.2	1.70±0.03	2.25	1356±32	37.1	62.9	27.0±0.7	41.7±0.1	11.9±0.2	17.9±0.5	9.1±0.1	--	--			2.9±0.09	4.5
Poplar	46.1±0.3	54.1	45.0	0.8	0.85±0.01	1.20	1036±46	53.9	46.1	20.1±0.9	31.7±0.7	16.0±0.4	10.0±0.4	10.1±0.5	--	--			12.8±0.28	16.8
Hi-S poplar	94.5±0.1	96.9	2.6	0.5	17.3±0.04	37.0	1612±36	5.5	94.5	33.6±0.7	70.7±0.1	2.5±0.1	32.0±0.7	1.6±0.1	--	--			1.4±0.07	3.1
Palm EFB	49.7±0.8	53.1	45.4	1.4	0.99±0.03	1.17	582±11	50.3	49.7	11.3±0.2	21.7±0.1	8.7±0.4	6.1±0.1	5.3±0.2	--	--			42.1±0.26	32.3
Switchgrass	12.7±0.1	24.3	72.8	2.9	0.15±0.01	0.34	543±9	87.3	12.7	10.0±0.2	8.3±0.1	10.8±0.1	1.4±0.1	8.5±0.1	4.93±0.05	5.64±0.06	53	60	53.1±0.83	49.6
Corn stover	22.8±2.5	46.3	50.3	3.3	0.30±0.04	0.89	391±43	77.2	22.8	7.3±0.8	8.4±0.1	6.2±0.2	1.8±0.1	5.5±0.8	7.88±0.06	7.42±0.12	48	63	101.5±0.89	158.6
Wheat straw	25.4±0.2	40.2	55.9	3.9	0.34±0.01	0.74	488±16	74.6	25.4	9.2±0.3	12.4±0.1	10.3±0.5	2.6±0.1	6.6±0.2	2.69±0.01	4.55±0.01	63	35	30.0±0.48	17.6

[a] Relative %S content by DFRC, on a mol% ∑SG basis.
[b] Relative %S content via HSQC NMR integration, on a mol% ∑SGH basis; from single samples (not replicated) simply to provide a comparison.
[c] Relative %G content via HSQC NMR integration, on a mol% ∑SGH basis; from single samples (not replicated) simply to provide a comparison.
[d] Relative %H content via HSQC NMR integration, on a mol% ∑SGH basis; from single samples (not replicated) simply to provide a comparison.
[e] Molar S/G from DFRC.
[f] Molar S/G from HSQC NMR.
[g] DFRC monomers yield from DFRC, on a µM/g of KL basis (as is the preferred method for thioacidolysis monomers reporting).
[h] DFRC monomers relative %G monomers, on a mol% of the total monomers ∑SG from DFRC. [%G + %S = 100%]
[i] DFRC monomers relative %S monomers, on a mol% of the total monomers ∑SG from DFRC. [%G + %S = 100%]
[j] DFRC total monomers ∑SG on a wt% KL basis
[k] Hydrogenolysis total S monomers ∑S, on a wt% KL/ basis. [∑G + ∑S = ∑SG in Table 1]
[l] Hydrogenolysis total G monomers ∑G, on a wt% KL/ basis. [∑G + ∑S = ∑SG in Table 1]
[m] DFRC total S monomers, S_{trans} + S_{cis}, ∑S on a wt% KL basis [∑G + ∑S = ∑SG by DFRC in this Table]
[n] DFRC total G monomers, G_{trans} + G_{cis}, ∑G on a wt% KL basis [∑G + ∑S = ∑SG by DFRC in this Table]
[o] *p*-Coumarate (*p*CA) hydrogenolysis product (methyl dihydro-*p*-coumarate) released by hydrogenolysis, on a wt% KL basis. [Note: actual product wt%, not on a *p*CA basis!] [Table 1 *p*CA + FA = ∑PA by hydrogenolysis]
[p] Ferulate saponification product, ferulic acid FA, released by saponification, on a wt% ∑PA basis. [Note: actual product wt%, not on an FA basis!] [*p*CA + FA = ∑PA by hydrogenolysis in Table1]
[q] Fraction of ferulate (%FA) hydrogenolysis product, on a wt% total hydrogenolysis PA (FA + *p*CA) basis.
[r] Fraction of ferulate (%FA) saponification product, on a wt% total saponification PA (FA + *p*CA) basis.
[s] Total hydrogenolysis phenolates (∑PA), on a mol% core-lignin monomers ∑GS (from Table 1) basis. [This can total >100% because *p*CA and FA also acylate polysaccharides in grass cell walls]
[t] Total NMR-determined phenolates (∑PA), on a mol% core-lignin monomers ∑HGS basis. [This can total >100% because *p*CA and FA also acylate polysaccharides in grass cell walls, and HSQC overestimates endgroups]
Note: ML-*p*HB conjugates were not measured by DFRC; as the *p*HB levels vary, and as *p*HB mainly acylates S-units, the willow/aspen/poplar/palms DFRC levels therefore track poorly with hydrogenolysis levels.
